# Ingroup solidarity drives social media engagement after political crises

**DOI:** 10.1073/pnas.2512765122

**Published:** 2025-08-28

**Authors:** Malia Marks, Yara Kyrychenko, Johan Gärdebo, Jon Roozenbeek

**Affiliations:** ^a^Department of Psychology, University of Cambridge, Cambridge CB2 3EB, United Kingdom; ^b^Cambridge Centre for History and Economics, University of Cambridge, Cambridge CB5 8AB, United Kingdom

**Keywords:** social media, US elections, social identity, solidarity, intergroup relations

## Abstract

Social media are often said to exacerbate polarization by platforming hostility between groups. However, positive social emotions like ingroup solidarity may also drive social media engagement, particularly after major threats such as military invasions or terror attacks. In this preregistered study, we examine the socioemotional drivers of engagement following group threats in the context of the US 2024 presidential campaign trail, where both major political parties faced crises in July of 2024. We test how ingroup solidarity and outgroup hostility predicted social media users’ engagement with 62,118 posts by 484 US partisan accounts before and after the first Trump assassination attempt (July 13) and Biden’s re-election campaign suspension (July 21). We find that, while outgroup hostility is typically the dominant predictor of engagement, interactions with ingroup solidarity surged among Republicans after the Trump shooting and among Democrats after Biden’s withdrawal. We show that negativity toward other groups is not always key to going viral. Rather, positive ingroup emotions appear to play a leading role in times of crisis.

Recent research has shown that negative emotions, moral outrage, and hostile mentions of social outgroups are associated with increased virality, especially in the highly polarized US context (1–3). Yet, the psychological factors driving online virality remain unclear (4). Empirical (5, 6) and theoretical (7, 8) literature in social psychology suggests that positive intergroup emotions, such as ingroup solidarity, take on greater importance after events presenting group threats. In this preregistered study, we test these possibilities for two competing and highly polarized groups: US Republicans and Democrats prior to the 2024 presidential election. In the span of just eight days, both parties experienced major crises, with then-candidate Donald Trump being shot in an assassination attempt on July 13, and then-president Joe Biden suspending his re-election campaign on July 21. By examining changes in the predictors of online engagement by party in response to these destabilizing events, we can better understand how, why, and when content goes viral online.

Social identity and self-categorization theories (9) posit that mere group categorization can produce a preference for the ingroup (i.e., ingroup solidarity or favoritism) and dislike of the outgroup (i.e., outgroup hostility or derogation). These theories have been used to explain affective polarization and its links to social media, particularly in the United States (2, 10). Social media has been hypothesized to exacerbate polarization by making group categorization salient, creating echo chambers, and amplifying morally outrageous, negative, and extreme content (4). However, this phenomenon appears to vary by geopolitical context (11). For instance, following a terror attack in France, social media posts contained more positive social emotions than negative affect (5). After Russia invaded Ukraine in 2022, ingroup solidarity became a dominant predictor of engagement with posts by Ukrainian social media accounts, while outgroup hostility became less engaging (6).

More broadly, much of the literature on intergroup discrimination indicates ingroup favoritism as the dominant force driving intergroup prejudice (7), while proponents of intergroup emotions theory suggest that ingroup solidarity should be more important than outgroup hostility during conflict or group threats in particular (8). For instance, the September 11 attacks were followed by the “rally-round-the-flag” effect, increasing support for the group leader (12). However, it is unclear whether this effect is isolated to extreme cases like mass attacks on civilian targets and military invasions, or if it might also occur after less severe threats, such as an attack targeting the group’s leader (e.g., the Trump assassination attempt) or even a nonviolent but unexpected loss of leadership (e.g., Biden’s campaign suspension). Moreover, it is unknown whether such an effect would manifest in the US context, where party identities are so deeply ingrained that pronounced in-party favoritism and out-party hostility is observed even when party membership is less salient (13).

To address these questions, we examine the changes in Facebook users’ engagement with posts from US conservative (i.e., Republican-aligned) and liberal (i.e., Democrat-aligned) Facebook accounts from the eight days before the attempted assassination of Donald Trump (on July 13) through the eight days after Biden’s withdrawal (on July 21). We find that engagement increased for posts containing solidarity but decreased for posts containing hostility for Republicans after July 13 and for Democrats after July 21. These findings highlight the role of positive group emotions in times of crisis, even in the midst of hostile competition with an outgroup. Contrary to previous findings (2), outgroup hostility is therefore not always the strongest predictor of engagement online.

## Results

**Fig. 1.**
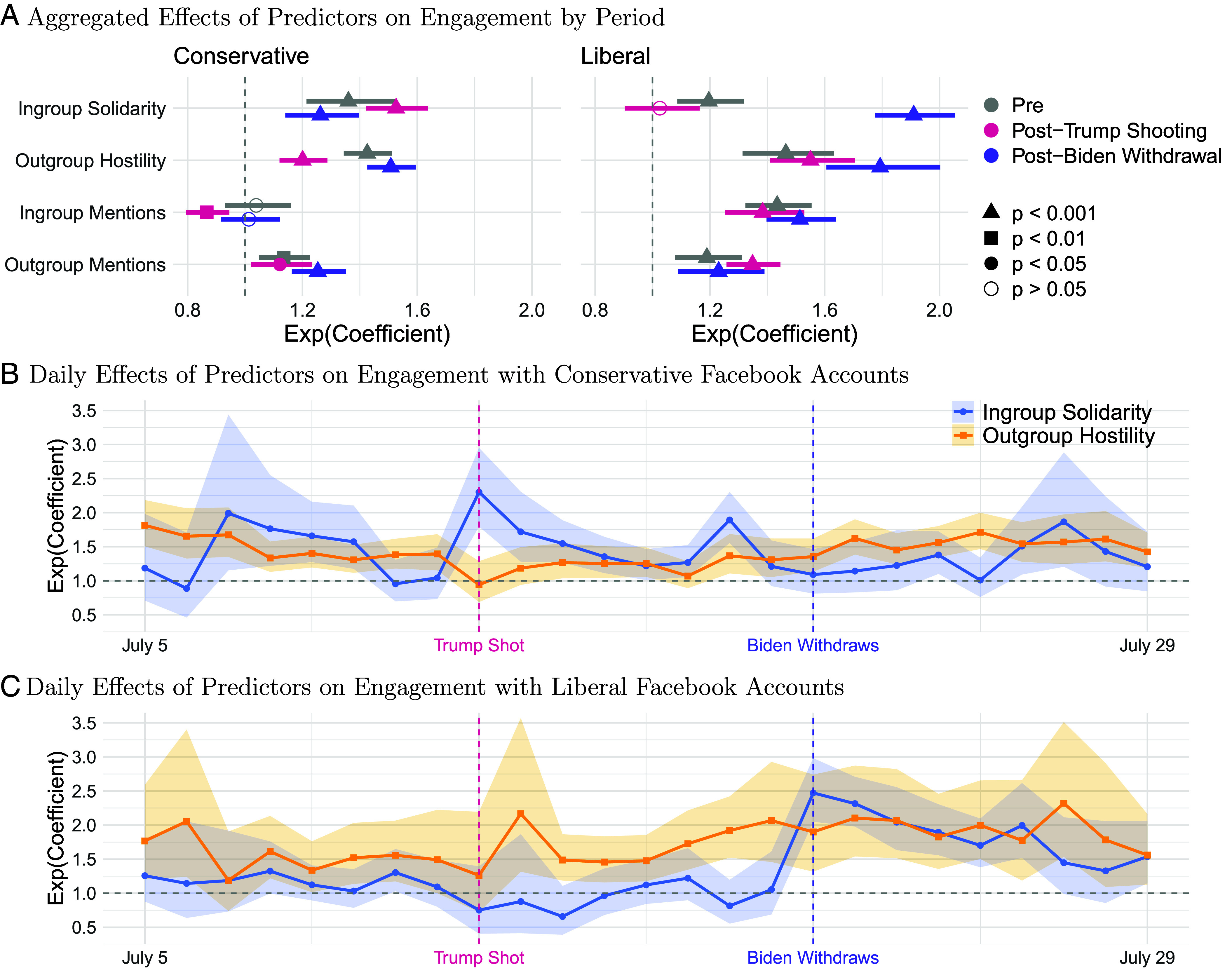
Aggregated effects of predictors on engagement by period (*A*) and daily effects of predictors on engagement with conservative (*B*) and liberal (*C*) accounts (N=29,198 conservative and N=32,920 liberal Facebook posts; $N_{\mathrm{total}}=62,118$). Dots represent exp(β) and whiskers or shaded areas are 95% CI.

We collected 62,118 public posts from 484 Facebook accounts run by US politicians and partisan commentators or media sources from July 5 to 29. After using GPT-4o to classify all posts as containing ingroup solidarity and outgroup hostility, we fit mixed-effects linear regressions subset by time period and political affiliation predicting log-transformed engagement, based on whether a post contained ingroup solidarity, outgroup hostility, and mentioned the ingroup or the outgroup, with a random effect of account name (following ref. 6). To assess how effects changed by period, we fit a regression for each political party with each independent variable interacting with the time period. Comparisons of effects across periods are reported in full in the supplement and summarized below, where we also report average effects for each period (Fig. 1). Power analyses conducted post hoc for the time-period comparison regression models (separately for conservative and liberal accounts) indicated that the available sample size was sufficient to detect a small effect (Cohen’s $f^{2}=0.02$) with 95% power at a significance level of 0.01. See *Materials and Methods* for details. Descriptively, conservative posts showed outgroup hostility 23.94% of the time, and ingroup solidarity 7.82% of the time. Of liberal posts, 5.13% showed outgroup hostility and 6.50% showed ingroup solidarity.

For conservatives, posts with ingroup solidarity received 36% more engagement than posts without in the pre-Trump-shooting-period (exp(β) = 1.36, 95%CI = [1.21,1.52], t(9674)=5.31, P<0.001), increasing significantly after Trump was shot (exp(β) = 1.53, 95%CI = [1.14,1.63], t(8705)=11.77, P<0.001), and significantly decreasing after Biden withdrew (exp(β) = 1.26, 95%CI = [1.14,1.39], t(10819)=4.49, P<0.001). Posts with outgroup hostility received 43% more engagement than those without in the preperiod (exp(β) = 1.43, 95%CI = [1.35,1.51], t(9674)=11.77, P<0.001) insignificantly diminishing after Trump was shot (exp(β) = 1.20, 95%CI = [1.11,1.28], t(8705)=5.17, P<0.001), then significantly increasing after Biden withdrew, exp(β) = 1.51, 95%CI=[1.42,1.60], t(10819)=14.39, P<0.001). Outgroup hostility was the dominant predictor of engagement with posts by Republicans in the pre-Trump-shooting and post-Biden-withdrawal periods, but ingroup solidarity was dominant after the Trump shooting.

For liberals, posts with ingroup solidarity received 20% more engagement than those without in the pre-Trump-shooting-period (exp(β) = 1.20, 95%CI = [1.08,1.32], t(10637)=3.64, P<0.001), not changing significantly but becoming an insignificant predictor after Trump was shot (exp(β) = 1.03, 95%CI = [0.90,1.16], t(10515)=0.39, P=0.70), then significantly rising after Biden withdrew (exp(β) = 1.91, 95%CI = [1.77,2.05], t(10637)=17.45, P<0.001). Posts with outgroup hostility saw 46% more engagement than those without during the preperiod (exp(β) = 1.47, 95%CI = [1.31,1.63], t(10637)=6.88, P<0.001), rising insignificantly after Trump was shot (exp(β) = 1.55, 95%CI = [1.40,1.70], t(10515)=9.02, P<0.001), and insignificantly increasing after Biden withdrew (exp(β) = 1.79, 95%CI = [1.60,1.99], t(11768)=10.38, P<0.001). Before Biden’s withdrawal, outgroup hostility was the dominant predictor of engagement on Democrat posts, with solidarity becoming the dominant predictor afterward.

## Discussion

Research on the drivers of online virality usually emphasizes hostility and negativity (1, 2) and often ignores relevant social context (6). We tested how engagement with posts by partisan accounts changes following disruptive political events from 2024: the July 13 Trump assassination attempt and the July 21 Biden campaign suspension. Although outgroup hostility was ordinarily the dominant predictor of engagement for both Republicans and Democrats, ingroup solidarity became significantly more predictive immediately following threats to group leadership, extending prior findings (6) to less severe and even nonviolent threats, as compared to major terror attacks and military invasions (5, 6). Notably, unlike prior work on engagement changes after the 2022 Russian invasion of Ukraine (6), the opposing groups in our study were not strictly responsible for the perceived threat—the political affiliation of Trump’s shooter remains unclear and Biden’s withdrawal was encouraged by Democrats and Republicans alike—indicating that this effect is likely due to threat to the ingroup rather than from the outgroup. Our findings add nuance to previous research indicating outgroup hostility as a leading driver of engagement (2), highlighting instead a more contextual understanding, with ingroup solidarity dominating in times of crisis. Conversely, our results indicate that outgroup hostility may receive more interactions when the opposing group is threatened, indicating a potential area for future study. Importantly, people engaging with social media posts may differ from the general public, leaving future work to examine whether social emotions follow similar patterns in offline interactions.

## Materials and Methods

This study was preregistered at AsPredicted #185461. Methodological details and additional preregistered analyses are described in *SI Appendix* (see OSF).

We collected all Facebook posts from July 5 to 29, 2024, by sitting American politicians, major partisan news outlets, and top political pundits using Facebook’s CrowdTangle (see OSF for list of accounts). We counted a post as mentioning ingroup or outgroup if it contained any words associated with ingroup or outgroup identity from ref. 2 and used GPT-4o as a binary classifier (14) for ingroup solidarity and outgroup hostility. Two human coders independently labeled 3,000 posts to assess GPT-4o accuracy, which was high (solidarity F1-macro = 0.79, hostility F1-macro = 0.81).

To test the main hypotheses, we fit mixed effects linear regressions for Republicans and Democrats for each time period: the pre- (July 5 to 12), post-Trump shooting (July 13 to 20), and post-Biden withdrawal periods (July 21 to 29). Models predicted log-transformed engagement per post (sum of all Facebook reactions, e.g., likes, shares, and angry reactions) with binary indicators of ingroup solidarity, outgroup hostility, and ingroup and outgroup mentions, with account name as a random effect (see ref. 6). Changes in effects between periods were compared using regressions with the same specifications but including interaction effects of time period with all fixed effects (*SI Appendix*).

## Supplementary Material

Appendix 01 (PDF)

## Data Availability

Anonymized data, code, our preregistration, and CSV data have been deposited in OSF (15).
