# Supplementary material for: Ingroup solidarity drives social media engagement after political crises
Source: Proc Natl Acad Sci U S A. 2025 Aug 28;122(35):e2512765122. doi: 10.1073/pnas.2512765122 (PMC12415245; doi:10.1073/pnas.2512765122)
Supplement: Supplementary file 1 — Appendix 01 (PDF) [file pnas.2512765122.sapp.pdf]

Supporting Information for

Ingroup Solidarity Drives Social Media Engagement After Political Crises

Malia Marks, Yara Kyrychenko, Johan Gärdebo, and Jon Roozenbeek

Corresponding Author: Malia Marks.
E-mail: mmm75@cam.ac.uk

This PDF file includes:

Supporting text
SI References

Supporting Information Text

Extended Methods

A. Data Collection. A full list of the Facebook pages included in our sample can be found in the supplementary information on this project's OSF page: https://osf.io/j8p7g/?view_only=49fdfb33d0074a1dafc469da015cf3bc. These included all sitting governors, senators, and representatives, major partisan news outlets according to AllSides Media Bias Chart as of August, 2024, and major liberal and conservative political influencers and commentators identified through Copilot queries.

A.1. Human Annotation. Two authors manually coded 3,000 posts for ingroup solidarity and outgroup hostility (binary). This included 500 randomly sampled posts per political affiliation per time period of the study. Inter-rater reliability was good. For posts by Democrats, ingroup solidarity showed an F1 Macro score of .85, Krippendorff's α of .69, and accuracy score of .97, whereas outgroup hostility showed an F1 Macro score of .86, Krippendorff's α of .71, and accuracy of .96. For posts by Republicans, ingroup solidarity showed an F1 Macro score of .98, Krippendorff's α of .86, and accuracy score of .93, whereas outgroup hostility showed an F1 Macro score of .96, Krippendorff's α of .85, and accuracy of .93.

A.2. Data Classification. We used GPT-4o with the below prompts to label the entire dataset. GPT-4o's performance compared to human labeling was good. For classifications of solidarity in Democrat posts, accuracy was .98, Krippendorff's α was .97, and the F1 Macro was .84. Solidarity in Republican posts showed an accuracy of .93, Krippendorff's α of .91, and an F1 Macro of .75. For Democrat hostility, accuracy was .97, Krippendorff's α was .96, and the F1 Macro was .83. Republican hostility showed an accuracy of .92, a Krippendorff's α of .90, and an F1 Macro of .87. Overall, GPT-4o showed good rates of agreement with human classifications of ingroup solidarity and outgroup hostility.

Ingroup solidarity prompt "You are a helpful assistant tasked with labeling whether a social media post from an American Democrat or Republican expresses solidarity with, or positive emotions towards, the poster's party, including specific party members, the whole party, or political allies in general. You should label posts as expressing ingroup solidarity with the poster's party only if they describe solidarity with or amongst party members, indicate liking of or pride in the poster's political allies, and/or mention the unity or strength or competency of the poster's political allies or party. This includes all posts where the poster praises the achievements or views of their party, talks about party members collaborating or supporting one another, or framing the poster's party as good, competent, popular, strong or moral people. A post expresses ingroup solidarity if it is directed at political allies, not if it is directed at political opponents or apolitical people. Take a moment to think, but only answer with 'yes' if the post expresses ingroup solidarity, or 'no' if it does not express ingroup solidarity. Label this post:"

Outgroup hostility prompt "You are a helpful assistant tasked with labeling whether a social media post from an American Democrat or Republican expresses hostility, or negative emotions, towards the opposing party (based on the poster's political affiliation), including specific party members, the whole party, or political opponents in general. You should label posts as expressing out-group hostility towards the opposing party only if they describe working against the opposing party, indicate disliking of political opponents, and/or mention the weakness or incompetency of the opposing party or political opponents. This includes all posts where the poster is critical of or disparages the work or views of the opposing party, or frames political opponents as bad, dangerous, incompetent, unpopular, extremist or immoral people. A post expresses out-group hostility if it is directed at political opponents, not if it is directed at the poster's own party or political allies. Take a moment to think, but only answer with 'yes' if the post expresses out-group hostility, or 'no' if it does not express out-group hostility. Label this post:"

A.3. Ingroup and Outgroup Mentions. Mentions of group identities were calculated based on liberal and conservative identity dictionaries used in (1).

Liberal identity mentions socialist*, communist*, marxist*, leftist*, liberal*, left-wing*, progressive*, social justice warrior, antifa, democrat*, dem, dems, libs.

Conservative identity mentions conservative*, gop, republican*, libertarian*, alt-right, right-wing, fascist*, far-right, far right, repub, repubs, maga.

A.4. Example posts.

Ingroup solidarity post from Democrat account "Deeply inspired by my friend and colleague Congresswoman Jennifer Wexton."

Ingroup solidarity post from Republican account "House Republicans will work to deliver accountability for the massive security failure that endangered the lives of President Trump and the attendees at the rally and tragically took the life of Corey Comperatore. This must never happen again."

Outgroup hostility post from Democrat account "OPINION | Republicans and right wing media outlets seem lost as they don't exactly know how to best hate Kamala Harris, writes Samantha Bee."

Outgroup hostility post from Republican account "Vice President Kamala Harris 'is not doing enough' to combat antisemitism, Rep. Buddy Carter told Newsmax, criticizing what he called her slow response to condemn the pro-Hamas, anti-war protest in Washington, D.C., this week."

Ingroup solidarity and outgroup hostility post from Democrat account "Over 1.5M women across Iowa woke up today with fewer rights. Iowa's new abortion ban is radical, dangerous, and another example of the hard-right taking us back in time. U.S. Senate Democrats will not stop fighting for abortion rights."

Ingroup solidarity and outgroup hostility post from Republican account "The 2017 Tax Cuts and Job Act isn't broken and doesn't need fixing. But many of its provisions are set to expire in 2025—and Democrats are threatening to let it happen, write Mike Pence and Pat Toomey"

Neither ingroup solidarity nor outgroup hostility post from Democrat account "Bee Taqueria has received the Michelin Bib Gourmand award for its exceptional and affordably priced elevated tacos."

Neither ingroup solidarity nor outgroup hostility post from Republican account "Karoline Leavitt, national press secretary for the Trump presidential campaign, shamed mainstream media outlets Friday night for suggesting former President Donald Trump wasn't hit by a bullet two weeks ago in Butler, Pennsylvania."

Descriptive Statistics for Hostility and Solidarity Of posts by conservatives before Trump was shot, 23.95% were coded as showing outgroup hostility; hostility was seen in 16.89% after the Trump shooting, and 24.21% after Biden dropped out. Solidarity was present in 4.64% of conservative posts before Trump was shot, 14.87% after Trump was shot, and 4.99% after Biden withdrew. 5.10% of posts by liberals were hostile before the Trump shooting, 5.67% after Trump was shot, and 4.69% after Biden withdrew. Solidarity was seen in 5.14% of liberal posts before Trump was shot, 3.40% after Trump was shot, and 10.51% after Biden withdrew.

B. Regression Analyses. As preregistered and drawing on the methodology from (2), we fit mixed effects linear regressions for each party and time period predicting log-transformed engagement based on binary indicators of ingroup solidarity, outgroup hostility, ingroup mentions, and outgroup mentions, with a random effect of account name. More formally:

$$\log(\text{Engagement}_{ij} + 1) = \beta_0 + \beta_1 \cdot \text{Solidarity}_{ij} + \beta_2 \cdot \text{Hostility}_{ij} + \beta_3 \cdot \text{IngroupMentions}_{ij} + \beta_4 \cdot \text{OutgroupMentions}_{ij} + u_j + \varepsilon_{ij}$$

$$u_j \sim \mathcal{N}(0, \sigma_u^2),$$
$$\varepsilon_{ij} \sim \mathcal{N}(0, \sigma^2).$$

Where:

- $\log(\text{Engagement}_{ij} + 1)$: log-transformed engagement for post i by account j,
- Solidarity_{ij}: binary indicator of ingroup solidarity,
- Hostility_{ij}: binary indicator of outgroup hostility,
- $\text{IngroupMentions}_{ij}$: binary indicator of mentioning the ingroup,
- $\text{OutgroupMentions}_{ij}$: binary indicator of mentioning the outgroup,
- u_j: random intercept for account j,
- ε_{ij}: residual error term.

To test whether the changes in coefficients based on the time period were significant, we fit the one mixed effects linear regression per party, with the same specifications as above but in addition including the interaction of every predictor with a categorical variable of period (pre, mid, or post), with mid as the reference level.

For Republicans, engagement with ingroup solidarity content increased significantly from the pre- into the mid-period ($\exp(\beta)$= 1.23, 95%CI= 1.08 - 1.39, $t(29198)$= 3.14, p= .002), then significantly degreased into the post-period ($\exp(\beta)$= 0.78, 95%CI= 0.69 - 0.88, $t(29198)$= -4.08, $p<$ .001). Engagement with outgroup hostility decreased insignificantly from the pre- into the mid-period ($\exp(\beta)$= 0.93, 95%CI= 0.85 - 1.01, $t(29198)$= -1.67, p= .096), and significantly increasing into the post-period ($\exp(\beta)$= 1.23, 95%CI= 1.13 - 1.34, $t(29198)$= 4.89, $p<$ .001).

For Democrats, engagement with ingroup solidarity content decreased insignificantly from the pre- to mid-period ($\exp(\beta)$= 0.94, 95%CI= 0.82 - 1.09, $t(32920)$= -0.76, p= .447), then increased significantly into the post-period ($\exp(\beta)$= 1.65, 95%CI= 1.45 - 1.88, $t(32920)$= 7.43, $p<$ .001). Engagement with outgroup hostility content rose insignificantly from the pre- into the mid-period ($\exp(\beta)$= 0.00, 95%CI= 0.87 - 1.15, $t(32920)$= -0.01, p= .995) and into the post-period ($\exp(\beta)$= 1.14, 95%CI= 0.00 - 1.31, $t(32920)$= 1.93, $p<$ .053).

We fit all models in R using the lme4 package. Upon visual inspection, the residuals did not significantly deviate from normality for all models. All Variance Inflation Factors in period-specific models were lower than 1.20, indicating an absence of multicollinearity among predictors. All p-values were estimated using Satterthwaite degrees of freedom.

The code for the main analyses is available in the Main Analyses R markdown and pdf files on our OSF.

Preregistered Analyses

Our preregistration can be found on OSF. Due to the limited length of the brief report format, we could not report all of our preregistered analyses in the main text. Analyses of rates of posting of ingroup and outgroup mentions, ingroup solidarity, and outgroup hostility, as well as analyses subset by poster account type, can be found in the Supporting Analyses R markdown file on our OSF. The posting rates and effects subset by account type generally reflect the patterns seen in our reported main analyses, with outgroup hostility decreasing and ingroup solidarity increasing following threats to posters' political party.

The effects of ingroup and outgroup mentions on engagement rates can be found in the Main Analyses R markdown file, as control variables in the main regression analyses. These factors are generally less predictive than ingroup solidarity and outgroup hostility.

Additionally, we intended to explore posts mentioning conspiracy theories about the attempted assassination of Trump. However, the dataset of 3000 posts that were coded by hand contained fewer than 10 examples of these theories. Because of the low rate of posts about these conspiracies, we opted not to move forward with that line of inquiry.

References

1. S Rathje, JJ Van Bavel, S van der Linden, Out-group animosity drives engagement on social media. *Proc. Natl. Acad. Sci.* **118**, e2024292118 (2021).
2. Y Kyrychenko, T Brik, S van der Linden, J Roozenbeek, Social identity correlates of social media engagement before and after the 2022 Russian invasion of Ukraine. *Nat. Commun.* **15** (2024).
